# Genome sequence of *Leishmania mexicana *MNYC/BZ/62/M379 expressing Cas9 and T7 RNA polymerase

**DOI:** 10.12688/wellcomeopenres.18575.2

**Published:** 2023-02-23

**Authors:** Tom Beneke, Ulrich Dobramysl, Carolina Moura Costa Catta-Preta, Jeremy Charles Mottram, Eva Gluenz, Richard J. Wheeler

**Affiliations:** 1 Sir William Dunn School of Pathology, University of Oxford, Oxford, UK; 2 Department of Cell and Developmental Biology, Biocentre, University of Würzburg, Würzburg, 97074, Germany; 3 Peter Medawar Building for Pathogen Research, Nuffield Department of Medicine, University of Oxford, Oxford, UK; 4 York Biomedical Research Institute, Department of Biology, University of York, York, UK; 5 Institute of Cell Biology, University of Bern, Bern, Switzerland

**Keywords:** Leishmania mexicana, Genome, Pilon polish

## Abstract

We present the genome sequence of *Leishmania mexicana* MNYC/BZ/62/M379 modified to express Cas9 and T7 RNA-polymerase, revealing high similarity to the reference genome (MHOM/GT2001/U1103). Through RNAseq-based annotation of coding sequences and untranslated regions, we provide primer sequences for construct and sgRNA template generation for CRISPR-assisted gene deletion and endogenous tagging.

## Introduction

*Leishmania mexicana* is a human-infective unicellular eukaryote and one of the species which cause leishmaniasis. It is commonly used as a model *Leishmania* species for molecular cell biology due to its lower virulence (causing cutaneous rather than visceral leishmaniasis) and its ability to readily differentiate into the amastigote form in appropriate axenic culture. We have previously described the generation of a genetically modified *L. mexicana* MNYC/BZ/62/M379 expressing Cas9 and T7 RNA polymerase as a strain enabling for rapid reverse genetic modifications ^1^. As this is not the reference genome strain (which is MHOM/GT/2001/U1103) ^2^ and may have accumulated mutations during laboratory culture and/or selection pressures of Cas9 or T7 expression, we sequenced the genome of this widely used strain as a high-quality reference for design of reverse genetic strategies.

## Methods

We have previously confirmed that these promastigotes are infectious to the sandfly vector ^3^. To ensure that the line was infectious to mammals, we infected an eight-week-old female BALB/c mouse footpad with stationary phase promastigotes (2.0 × 10 ^6^); after four weeks we purified amastigotes from the excised resulting lesion, which were then back-transformed to promastigotes in axenic culture in M199 supplemented with 20% FCS and 50 µg/ml gentamycin (Roche) and grown for seven passages. This gave rise to the cell line *L. mex* Cas9 T7 M. Genomic DNA from before and after mouse passage was extracted using phenol-chloroform DNA extraction as previously described ^4^. For Illumina sequencing, isolated DNA was diluted in 300 µL resuspension buffer (from Illumina TruSeq Nano DNA Library kit) and sonicated for nine (9) minutes using a Bioruptor 300 (Diagenode, set to low [0.5 interval]). The resulting 600 bp DNA fragments were processed for library construction using the TruSeq Nano DNA Library kit (Illumina). Sequencing followed on an Illumina NextSeq550 in paired-end mode (2×150nt) using a NextSeq 500/550 Mid Output 300 Cycles v2 Kit (Illumina, v2 kits now discontinued). 24,656,567 (7.4 Gb) and 26,015,449 reads (7.8 Gb) from before and after passage were obtained respectively. For Nanopore sequencing, a library was constructed from the after-passage sample (not additionally sonicated) using the 1D ^2^ (SQK-LSK309) kit and sequenced on a MinION (FLO-MIN106 flow cell), obtaining 329,692 1D and 48,711 1D ^2^ reads (total, 2.3 Gb; mean length, 6.1 kb; read *N*_50_, 8.0 kb; longest read, 448 kb).

We generated a trial *de novo* MNYC/BZ/62/M379 Cas9/T7 assembly using the Nanopore reads. Following adapter trimming using Porechop v0.2.4 we used a minimap2/racon/miniasm pipeline (v2.17-r974, v1.4.20 and v0.3-r179 respectively) ^5– 7^, then polished the assembly by mapping the adapter-trimmed (using TrimGalore! v0.6.0) ^8^ Illumina reads using BWA-MEM v0.7.17 ^9^ and 10 rounds of polishing using Pilon v1.23 ^10^ (total length, 31.4 Mb; contigs, 109; mean length, 291 kb; *N*_50_, 640 kb; longest contig, 2.87 Mb). Synteny inspection using SyMAP ^11^ in comparison to the MHOM/GT/2001/U1103 reference genome ^2^ showed one (1) or two (2) contigs per chromosome (except for chromosomes 34, four (4) contigs; 19, three (3) contigs; 10, three (3) contigs; 8, four (4) contigs) and no evidence for chromosomal segmental deletion or duplication.

To simplify genome annotation, we therefore opted to polish the MHOM/GT/2001/U1103 genome (NCBI Genome Assembly GCA_000234665.4) with the Illumina reads to generate a MNYC/BZ/62/M379 Cas9/T7 genome instead of using the *de novo* assembly. Following adapter trimming using TrimGalore! V0.6.0 (default settings) and removal of unfixable reads, the genome was polished by mapping the Illumina reads to the genome using BWA-MEM v0.7.17 ^9^ and one (1) round of polishing using Pilon v1.23 fixing SNPs and indels ^10^, identifying 21500 SNPs, 3828 small insertions and 4878 small deletions (as defined by Pilon ^10^; mean insertion length 2.25 bp, mean deletion length 3.01 bp). The resulting updated and annotated genome sequence is available as a supplement ^12^. Pilon, run in changes mode, identified only 193 SNPs and no changes in coding sequences following mouse passage. Note that neither T7 nor Cas9 are present in this polished genome as Cas9 is not chromosomally integrated (instead expressed from an episome ^1^) and T7 is integrated into the highly repetitive 18S rRNA array which is collapsed in the reference genome.

Aneuploidy is known to be common among *Leishmania* ^2^. Indeed, sequencing read coverage (how many times each nucleotide was sequenced) was not uniform across all chromosomes. Average read coverage throughout most chromosome was 144±5 (mean±sd.; diploid) excluding three outlier chromosomes: chromosome 3 (coverage, 218; triploid), 16 (coverage, 214; triploid) and 30 (coverage, 277; tetraploid).

Updated MHOM/GT/2001/U1103 genome annotations were prepared from existing resources, then transferred to MNYC/BZ/62/M379 Cas9/T7 accounting for coordinate changes due to indels. MHOM/GT/2001/U1103 ORFs and non-coding RNAs from TriTrypDB v50 ^13^ were taken as the start set. Previous RNAseq analysis ^14^ (BioProject accession number PRJEB8829) mapped spliced leader acceptor sites (SLASs, the site of trans splicing of a leader sequence common to all processed mRNAs) and polyadenylation sites (PASs) which define the bounds of the mRNA, from which suggested gene extensions and truncations were listed. We included these changes when a valid ORF (with a start and stop codon and no internal stops) was retained and mapped the 5’ and 3’ UTR based on the most commonly observed SLAS and PAS for each gene respectively. Previously identified novel genes with a valid ORF and evidence for expression as a polyadenylated transcript ^13^ were also included. To distinguish these gene models from the reference genome annotation we prefixed the gene names with “LmxM379c” indicating the strain name and its expression of Cas9 and T7.

The Cas9 enables CRISPR-assisted genome editing, while the T7 RNA polymerase allows use of sgDNA encoding a T7 promoter and sgRNA to program Cas9 activity. Using our previously published ‘LeishGEdit’ pipeline ^1^ and our updated primer design software ^15^ that is based on the CCTop CRISPR/Cas9 Target Prediction Tool ^16^, we designed primers for: 1) PCR-based generation of constructs for endogenous protein tagging (uf/ur primers for N terminal tagging or df/dr primers for C terminal tagging, using the pPOT, pLPOT and pPLOT series of plasmids) and gene deletion (uf/dr primers, using the pT series). 2) PCR-based generation of sgRNA templates for tagging and deletion (5g/3g primers for deletion, 5g primer for N terminal tagging and 3g primer for C). 3) Primers within each protein-coding gene ORF validation of gene deletion by diagnostic PCR (vf/vr primers) (based on the Primer3 primer design software ^17^). We also designed uf primers carrying a unique 17-nt DNA barcode ^15^ for generating barcoded pools of deletion mutants.

As this set of primers accounts for strain-specific SNPs and indels we recommended them as a standardised ‘first attempt’ for tagging and deletion genes in *L. mex* Cas9 T7 M, and we will be using them for future high-throughput reverse genetic analyses.

### Ethics statement

All experiments were conducted according to the Animals (Scientific Procedures) Act of 1986, United Kingdom, and had approval from the University of York Animal Welfare and Ethical Review Body (AWERB) committee. All efforts were undertaken to minimise the suffering of animals.

## Data Availability

BioProject: Leishmania mexicana MNYC/BZ/62/M379 Cas9/T7 whole genome sequencing; Accession number: PRJNA853937, https://identifiers.org/bioproject:PRJNA853937 ^18^ Sequence Read Archive: Next generation sequencing of Leishmania mexicana Cas9/T7 strain: after mouse passage (SRR19895146). Accession number: SRR19895146; https://identifiers.org/insdc.sra:SRR19895146 ^19^ Sequence Read Archive: Nanopore sequencing of Leishmania mexicana Cas9/T7 strain (SRR20123517). Accession number: SRR20123517; https://identifiers.org/insdc.sra:SRR20123517 ^20^ Zenodo: Gene tagging and gene deletion resources for Leishmania mexicana MNYC/BZ/62/M379 Cas9/T7 strain, https://doi.org/10.5281/zenodo.7313190 ^12^ This project contains the primer sequences, barcodes and the GFF file containing the sequence and the annotations of the *L. mexicana* MNYC/BZ/62/M379 T7/Cas9.

## Analysis code

All code for genome assembly, polishing, annotation updates and annotation transfer are available from GitHub: https://github.com/Wheeler-Lab/genome-lmexcas9t7/tree/v1.0.1; and archived in Zenodo: https://doi.org/10.5281/zenodo.7357174 ^21^

License: GNU GPL-3.0

### Reporting guidelines

Zenodo: ARRIVE E-10 Checklist for “Genome sequence of *Leishmania mexicana* MNYC/BZ/62/M379 expressing Cas9 and T7 RNA polymerase”, https://doi.org/10.5281/zenodo.7330926 ^22^.

Data are available under the terms of the Creative Commons Attribution 4.0 International license (CC-BY 4.0).
