# Supplementary material for: Genome sequence of Leishmania mexicana MNYC/BZ/62/M379 expressing Cas9 and T7 RNA polymerase
Source: Wellcome Open Res. 2023 Feb 23;7:294. Originally published 2022 Dec 5. [Version 2] doi: 10.12688/wellcomeopenres.18575.2 (PMC9975418; doi:10.12688/wellcomeopenres.18575.2)
Supplement: Peer review history

# Peer review: Reviewer response for version 2

**DOI:** 10.21956/wellcomeopenres.21134.r55188

**Published:** 2023-02-23

**Authors:** Richard McCulloch

**Affiliations:** 1 Wellcome Centre for Integrative Parasitology, School of Infection and Immunity, University of Glasgow, Glasgow, UK

The authors have more than adequately addressed the relatively minor issues raised.

Are sufficient details of methods and materials provided to allow replication by others?

Partly

Is the rationale for creating the dataset(s) clearly described?

Yes

Are the datasets clearly presented in a useable and accessible format?

Yes

Are the protocols appropriate and is the work technically sound?

Yes

Reviewer Expertise:

Genome biology of trypanosomatid parasites

I confirm that I have read this submission and believe that I have an appropriate level of expertise to confirm that it is of an acceptable scientific standard.

# Peer review: Reviewer response for version 1

**DOI:** 10.21956/wellcomeopenres.20597.r53618

**Published:** 2023-01-12

**Authors:** Stephen M. Beverley

**Affiliations:** 1 Department of Molecular Microbiology, Washington University School of Medicine, St. Louis, MO, USA

This is a fairly straightforward work providing the genome sequence of a workhorse Leishmania mexicana strain in common use for CRISPR genome editing studies. It will be a useful resource to many laboratories.

 The authors might comment upon the 'clonality' of this line - in past work from these investigators the parasites were not always of clonal origin and hence some heterogeneity may exist which could confound the analysis. Were the parent lines clonal, and since their origin, how many cell doublings have occurred that might further introduce heterogeneity?

 Secondly, the use of triploid or tetraploid for specific chromosomes is incorrect - it should be trisomic or tetrasomic.

Are sufficient details of methods and materials provided to allow replication by others?

Yes

Is the rationale for creating the dataset(s) clearly described?

Yes

Are the datasets clearly presented in a useable and accessible format?

Yes

Are the protocols appropriate and is the work technically sound?

Yes

Reviewer Expertise:

Leishmania molecular genetics

I confirm that I have read this submission and believe that I have an appropriate level of expertise to confirm that it is of an acceptable scientific standard.

# Peer review: Reviewer response for version 1

**DOI:** 10.21956/wellcomeopenres.20597.r53621

**Published:** 2023-01-04

**Authors:** Richard McCulloch

**Affiliations:** 1 Wellcome Centre for Integrative Parasitology, School of Infection and Immunity, University of Glasgow, Glasgow, UK

In this article, Beneke and colleagues provide an extremely useful resource: the genome sequence of a Leishmania mexicana strain expressing Cas9 and T7 polymerase, which is widely used to allow for CRISPR genome editing. The main focus of the article is in understanding if these modifications of an existing and sequenced 'reference' L. mexicana strain (to allow CRISPR editing) have resulted in undetected genome changes, including sequence variations that might reduce the effectiveness of primers based on the reference genome. The data suggest no large scale genome changes have occurred, but that some SNPs should be accounted for in designing primers for CRISPR-based experiments. As such, the study and new genome are cleary of considerable value. Nonetheless, some methods could be more clearly explained.

  

1. Are the authors correct when they state that they generated 734 Gb and 7.8 Gb of sequence from before and after passage? This difference appears at odds with the very similar number of reads recovered from the two samples: 24,656,567 and 26,015,449.
2. In the article, the authors suggest that they used Pilon v1.23 to 'polish the [reference] MHOM/GT/2001/U1103 genome'. I would suggest this phrasing is perhaps confusing and could be improved. Pilon is an algorithm that can be used to improve an existing genome assembly or identify differences between related assemblies. In the context of the de novo assembly of the MNYC/BZ/62/M379 Cas9/T7 genome, 'polishing' appears appropriate, since the Illumina reads improve the Nanopore assembly. However, it appears that the approach taken when comparing the MNYC/BZ/62/M379 Cas9/T7 Illumina reads to the MHOM/GT/2001/U1103 genome assembly has not 'polished' (i.e. altered) the latter genome, but instead identified differences between them.
3. In describing the differences between the two genomes, what do the authors mean by 'small' insertions and deletions; can they specify the size range?
4. The following sentence is somewhat confusing:
  'Indeed, chromosomal coverage was not uniform, coverage was 144±5 (mean±sd.) excluding three outliers: chromosome 3 (coverage, 218; triploid), 16 (coverage, 214; triploid) and 30 (coverage, 277; tetraploid).' What is the 'coverage' they refer to; and do they mean that coverage only deviated from relatively uniform levels in the three chromosomes mentioned, or are they suggesting there may be further ploidy variation (not discussed)?

Are sufficient details of methods and materials provided to allow replication by others?

Partly

Is the rationale for creating the dataset(s) clearly described?

Yes

Are the datasets clearly presented in a useable and accessible format?

Yes

Are the protocols appropriate and is the work technically sound?

Yes

Reviewer Expertise:

Genome biology of trypanosomatid parasites

I confirm that I have read this submission and believe that I have an appropriate level of expertise to confirm that it is of an acceptable scientific standard, however I have significant reservations, as outlined above.

# Peer review: Reviewer response for version 1

**DOI:** 10.21956/wellcomeopenres.20597.r53617

**Published:** 2022-12-23

**Authors:** José María Requena

**Affiliations:** 1 Centro de Biología Molecular Severo Ochoa (CSIC-UAM), Departamento de Biología Molecular, Instituto Universitario de Biología Molecular (IUBM), Universidad Autónoma de Madrid, Madrid, Spain

In this article, Beneke *et al. *inform on the genomic sequencing and genome assembly for the *Leishmania mexicana* MNYC/BZ/62/M379 strain, which was manipulated to express CRISPR-associated protein 9 (Cas9) and T7 RNA polymerase. The creation of this cell line represents the starting point (and essential step) to apply the successful methodology of CRISPR-assisted gene deletion and endogenous tagging that was developed previously by these authors. Although the genome sequence for other *L. mexicana* strain (MHOM/GT/2001/U1103) is publically available, it is necessary to know the exact nucleotide sequence for the genes in the particular Leishmania line in which CRISPR methodology will be used. As mentioned by the authors the *L. mexicana* MNYC/BZ/62/M379 strain is widely used in different laboratories.

 The methodological procedures used for generating the raw sequencing data are described in a detailed manner. Also, the bioinformatics procedures used for genome assembly and sequence polishing are clearly described. Apart from generating the genome sequence, they conducted genome annotations and transcripts delineation (including UTRs). As another valuable resource, the authors have designed oligonucleotide sequences to accomplish gene tagging or deletion of every gene in *L. mexicana* MNYC/BZ/62/M379.

 In sum, this article contains valuable datasets. In this regard, this reviewer acknowledges the effort made by the authors to generate these valuable resources and encourages users to inform about possible inconsistencies in the information provided, as an effective way to improve and curate genome annotations between all.

Are sufficient details of methods and materials provided to allow replication by others?

Yes

Is the rationale for creating the dataset(s) clearly described?

Yes

Are the datasets clearly presented in a useable and accessible format?

Yes

Are the protocols appropriate and is the work technically sound?

Yes

Reviewer Expertise:

Molecular Biology, Leishmania, NGS, genomics, transcriptomics

I confirm that I have read this submission and believe that I have an appropriate level of expertise to confirm that it is of an acceptable scientific standard.
